# lncRNA NONRATT013819.2 promotes transforming growth factor-β1-induced myofibroblastic transition of hepatic stellate cells by miR24-3p/*lox*

**DOI:** 10.1515/med-2022-0460

**Published:** 2022-04-04

**Authors:** Can-Jie Guo, Qin Pan, Xiong Ma

**Affiliations:** Division of Gastroenterology and Hepatology, Key Laboratory of Gastroenterology and Hepatology, Ministry of Health, Renji Hospital, School of Medicine, Shanghai Jiao Tong University, Shanghai Institute of Digestive Disease, Shanghai 200001, China; Research Center, Shanghai University of Medicine and Health Sciences Affiliated Zhoupi Hospital, Shanghai, 201318, China; Digestive Disease Laboratory and Department of Gastroenterology, Xinhua Hospital, School of Medicine, Shanghai Jiaotong University, 1665 Kongjiang Road, Shanghai 200092, China; Division of Gastroenterology and Hepatology, Key Laboratory of Gastroenterology and Hepatology, Ministry of Health, Renji Hospital, School of Medicine, Shanghai Jiao Tong University, Shanghai Institute of Digestive Disease, 145 Middle Shandong Road, Shanghai 200001, China

**Keywords:** liver fibrosis, NONRATT013819.2/miR24-3p/lox, ECM reconstruction, cytoskeleton markers, proliferation, apoptosis, cell cycle

## Abstract

Long noncoding RNAs (lncRNAs) are key regulators of hepatic stellate cells (HSCs), yet the role of upregulated lncRNA-NONRATT013819.2 in activated HSCs remains uncertain. In this study, the effects of NONRATT013819.2 on proliferation, apoptosis, migration, and contraction of transforming growth factor (TGF)-β1-induced HSCs were investigated. The mechanisms of NONRATT013819.2 on the activated HSCs were explored by loss-of-function of NONRATT013819.2 and gain-of-function of the target gene. Here, TGF-β1 treatment resulted in a gradual increase in the expression of cytoskeleton markers (collagen, α-SMA, and TIMP1), NONRATT013819.2, miR24-3p, and lysyl oxidase (Lox) over time in HSCs. NONRATT013819.2 acted as a sponge of miR24-3p to competitively abolish the inhibition of the *lox* gene in HSCs. Silencing of NONRATT013819.2 suppressed the expression of cytoskeleton markers, proliferation, and the proportion of cells that entered the S-phase, and promoted apoptosis in TGF-β1-activated HSCs. These effects were reversed when *lox* overexpression was introduced simultaneously. Similarly, silencing of NONRATT013819.2 also blocked ECM reconstruction, while recused by *lox* overexpression in TGF-β1-activated HSCs. In conclusion, upregulation of NONRATT013819.2 promotes the myofibroblastic transition by competitively binding miR24-3p to release *lox* in HSCs. Therefore, targeted therapy of NONRATT013819.2 may have the potential for liver fibrosis.

## 1 Introduction

Hepatic stellate cells (HSCs), which are located in the Disse gap between the hepatocyte plate and hepatic sinus, play an important role in the pathogenesis of liver fibrosis [1]. In normal liver, quiescent HSCs have weak relaxation and contraction. However, when there is liver damage, HSCs transition from a quiescent state to an activated state and the activated HSCs acquire the migratory, proliferative, and myofibroblast-like phenotype [2]. This myofibroblastic transition process leads to the accumulation of myofibroblasts, which contributes to the formation of liver fibrosis and the reconstruction of intrahepatic structures by secreting extracellular matrix (ECM) [3]. However, the mechanisms of HSC phenotypic transformation remain poorly understood.

Long noncoding RNAs (lncRNAs) are a novel class of ncRNAs with transcripts containing more than 200 nucleotides [4]. Current studies indicate that lncRNAs serve as activators or suppressors of HSCs to participate in the occurrence and development of myofibroblastic transition. For instance, lncRNA NEAT1-induced autophagy and activation of HSCs in mice [5]. To explore the differential expression profile of lncRNA during HSC myofibroblastic transition, we previously performed high-throughput sequencing in primary quiescent and activated HSC of rats. We found that the expression level of lncRNA NONRATT013819.2 was significantly upregulated more than fivefold in activated HSCs compared to the quiescent HSCs [6]. Meanwhile, the expression of NONRATT013819.2 was positively correlated with that of the lysyl oxidase (*lox*) gene, which is adjacent to each other at the chromosomal location [6]. However, whether NONRATT013819.2 and *lox* are involved in HSC myofibroblastic transition and the possible underlying mechanism remains unclear.

It is well known that miRNAs initiate the degradation of mRNA by binding to miRNA response elements (MREs) of mRNA with the attendant inhibition of the translation of target genes [7]. Numerous studies have found that the same MREs also exist on lncRNA and circRNA. Hence, different types of RNA containing MREs can “communicate” with one another simply by competing for the shared miRNAs, acting as ceRNAs, through the formation of lncRNA–miRNA–mRNA or circRNA–miRNA–mRNA ceRNA regulation networks [8]. This hypothesis is widely adopted when studying the role of lncRNA in disease pathogenesis. However, whether the relationship between NONRATT013819.2 and *lox* depends on ceRNA remains unclear.

In this study, we performed an overall analysis of the effects of NONRATT013819.2 on the biological properties of HSCs and investigated its mechanism. Our findings may improve the understanding of the myofibroblastic transition of HSCs and provide a novel therapeutic strategy against the activation and progression of liver fibrosis.

## 2 Materials and methods

### 2.1 Isolation, culture, and treatment of rat HSCs

Primary HSCs were isolated from three normal male Sprague–Dawley rats (weighing 400–500 g) by *in situ* perfusion and density gradient centrifugation using a modification of the methodology previously described by Friedman et al. [9]. Briefly, animals were anesthetized with ether and treated with 0.1 cc heparin sodium (1 mL/kg) via direct inferior *vena cava* injection immediately before perfusion. Subsequently, the serial infusion was carried out via rodent portal vein using D-Hank’s solution and then perfusion medium (Hank’s medium with 0.5 g/L collagenase IV and 1 g/L pronase E). Afterward, the isolated rat liver was cut into pieces and exposed to re-digestion using collagenase IV and DNase. Finally, the rat HSCs were isolated from cell suspension by density gradient centrifugation using 180 g/L Nycodenz (Sigma-Aldrich, St. Louis, MO, USA). Activation of HSCs was induced by the transforming growth factor (TGF)-β1 at a concentration of 10 ng/mL, and the cells were cultured for 0, 12, 24, 48, 72, and 96 h. All the materials for HSC isolation, culture, and identification were obtained from commercial sources. The rats received humane care according to the Guideline for the Care and Use of Laboratory Animals of the Chinese Academy of Sciences. Ethical approvals were obtained from the Ethics Committee of Renji Hospital, School of Medicine, Shanghai Jiao Tong University (Approval No. 82170615).

### 2.2 Construction of vectors

The following steps were involved in the construction of a silencing vector of lncRNA NONRATT013819.2. First, we designed a specific siRNA sequence that complementarily binds to NONRATT013819.2. Then, a complementary shRNA-DNA sequence with a hairpin structure was obtained according to the siRNA sequence. Next, the shRNA-DNA sequences were cloned into the linear pSIH1-H1-copGFP shRNA vector (System Biosciences, Palo Alto, CA, USA) to obtain lncRNA NONRATT013819.2 silencing vector (tagged as pSIH1-shRNA-NONR). Meanwhile, an invalid siRNA sequence was used as a negative control (NC) and tagged as pSIH1-NC. Finally, DNA sequencing was used to confirm the constructed vectors (pSIH1-shRNA-NONR and pSIH1-NC). All the primers are shown in Table S1.

Construction of the overexpression vector of *lox*. The coding sequence of rat *lox* (NM_017061.2, 1808 bp) was amplified using PCR, which contained an *Eco*RI cutting site, Kozak sequence (5′-GCCACC-3′], and a *Bam*HI cutting site. The product of PCR amplification was digested and cloned into a pcDH1-GFP lentiviral expression vector (System Biosciences). The recombinant vector was tagged pcDH1-lox and confirmed by DNA sequencing. Empty vectors were used as controls. All the primers are listed in Table S1.

### 2.3 Lentivirus packaging and infection

Transfection was carried out when the cells were in the logarithmic growth phase. Here, 2 µg recombinant vector (pSIH1-shRNA-NONR, pSIH1-NC, and pcDH1-lox) and 10 µg of pPACK™ Packaging Plasmid Mix (System Biosciences) were co-transfected into cells using diluted Lipofectamine 2000 (2 µL was diluted with 50 µL OPTI-MEM; Invitrogen, Carlsbad, CA, USA). After a 48-h culture, the supernatant was harvested and cleared by centrifugation at 5,000×*g* at 4°C for 5 min and then filtered by a 0.45 µm polyvinylidene difluoride membrane (Millipore, Billerica, MA, USA). The titer of the virus was determined by gradient dilution. Furthermore, the established lentiviruses with the sequences of shRNA-NONR, lox, and NC were tagged Lv-shRNA-NONR, Lv-lox, and Lv-NC, respectively.

Thereafter, HSCs cell line of lncRNA NONRATT013819.2 silencing and Lox overexpression were produced by lentiviral infection using the aforementioned packaged lentiviruses of Lv-shRNA-NONR and Lv-lox, respectively. The lentiviral infection was conducted when HSCs were in the logarithmic growth phase on six-well plates at 5 × 10^5^ cells/well. The viral solution was added at a multiplicity of infection (MOI) of 10. Infection efficiency was assessed by GFP fluorescence after infection for 72 h by a fluorescence inverted microscope (IX71, Olympus, Japan).

### 2.4 Analysis of RT-qPCR

Total RNA was isolated using the TRIzol^®^ Reagent (Invitrogen; Thermo Fisher Scientific). RT-qPCR was performed in triplicate for each sample. The relative amounts of NONRATT013819.2 and Lox were normalized against β-actin and miR24-3p against U6 snRNA. The fold change for each gene was calculated by the 2^−ΔΔCt^ method. The primers used are shown in Table S1.

### 2.5 Western blotting

Total protein was extracted from the cells using the M-PER mammalian protein extraction reagent (Pierce, Rockford, IL, USA). Equal quantities of protein (20 µg per lane) estimated by the bicinchoninic acid protein assay kit (Pierce) were loaded onto 11% SDS-PAGE and transferred onto nitrocellulose membranes. The blots were probed with a monoclonal antibodies against human collagen I (1:200, Cat#ab138492, Abcam, UK), α-SMA (1:500, Cat#ab5694, Abcam), tissue inhibitor of metalloproteinase (TIMP)1 (1:600, Cat#ab211926, Abcam), Lox (1:250, Cat#ab174316, Abcam), and β-actin (1:1200, Cat#ab8226, Abcam), followed by secondary horseradish peroxidase-conjugated antirabbit antibody (1:10,000, Cat#ab97080, Abcam). After washing, the bands were detected by chemiluminescence and imaged with X-ray films. β-Actin was used as an endogenous reference for normalization.

### 2.6 Luciferase activity assay

The current study utilized Targetscan7.1 (http://www.targetscan.org/) to predict the binding site of miR24-3p on the 3′ untranslated region (UTR) of Lox mRNA, and the binding sites of miR24-3p on NONRATT013819.2. Primers that targeted the 3′ UTR of the *lox* gene were designed such that flanking *Xba*I restriction sites were introduced into the 228-bp PCR product containing the miR24-3p target site (5′-TGAGCC-3′]. The primer sequences of PCR are shown in Table S1. PCR product was digested with *Xba*I (Takara Bio) and cloned into the pGL3-promoter luciferase reporter vector (Promega, Madison, WI, USA) to generate the vector pGL3-wild type (wt)-Lox. The miR24-3p target site in the pGL3-wt-Lox vector was mutated from 5′-TGAGCC-3′ to 5′-GCGCTA-3′ to construct the mutated reporter vector, tagged as pGL3-mt-Lox, using a Site-Directed Mutagenesis Kit (Takara Bio). The products of the vectors were confirmed by DNA sequencing. Endotoxin-free DNA samples were prepared in all cases. The miR24-3p mimic, the miR24-3p inhibitor, and miR24-3p NC were all synthesized by Invitrogen (Thermo Fisher Scientific) and then co-transfected with pGL3-wt-Lox or pGL3-mt-Lox into 293 cells. The sequences are shown in Table S1. Luciferase activity was measured using the dual-luciferase reporter assay system (Promega) after transfection for 48 h. Moreover, the effect of NONRATT013819.2 depletion on the luciferase activity of miR24-3p mimics was also observed in activated HSCs.

### 2.7 Cell Counting kit-8 assay

The HSCs viability was examined using Cell Counting Kit-8 (CCK-8; Dojindo, Kumamoto, Japan). After viral infection of HSCs for 48 h, HSCs were induced by 10 ng/mL TGF-β1 for 72 h, and then, the cells were harvested for the assay. Each sample was set with six repeating wells. The HSCs were seeded in a 96-well plate at a density of 2 × 10^3^ cells/well and cultured for 24 h. Then, 10 µL of CCK-8 reagent 8 was added to each well and cultured for another 12, 24, 48, and 72 h. Absorbance was measured at 450 nm on a microplate reader (Molecular Devices, Tokyo, Japan).

### 2.8 Flow cytometry

The cell cycle distributions and apoptosis rates of HSCs were measured by flow cytometry on fluorescence-activated cell sorting (FACS) Calibur (BD Biosciences, Franklin Lakes, NJ, USA) as previously described [10]. After viral infection of HSCs for 48 h, HSCs were induced by 10 ng/mL TGF-β1 for 72 h, and then, the cells were harvested for assay. Freshly isolated HSCs were fixed and then stained with propidium iodide followed by FACS analysis.

### 2.9 Gel contracture experiment

After viral infection of HSCs for 48 h, HSCs were induced by 10 ng/mL TGF-β1 for 72 h, and then, the cells were harvested for contraction assay on collagen gel. Rat-tail collagen (4 g/L; BD Biosciences) was preplated in 12-well plates and cultured for 1 h at 37°C to allow for gelatinization. Next, HSCs were plated on top of the gel (the density of cells and volume of gel were 1 × 10^6^ cells/well and 50 μL, respectively) and co-cultured for 48 h. Experiments were terminated by adding 53% ethanol. Finally, gels were imaged, and the collagen gel contraction was observed based on the area of the gels.

### 2.10 Immunofluorescence staining

The α-SMA expression of HSCs was evaluated by immunofluorescence (IF) staining. HSCs were harvested and washed with phosphate-buffered solution and then fixed in 4% paraformaldehyde. Next, HSCs were permeabilized with 0.5% Triton X-100 and blocked by 5% bovine serum albumin. Then, HSCs were incubated with monoclonal antibodies specific for α-SMA (1:100; Sigma-Aldrich, St. Louis, MO, USA) at 4°C for overnight followed by incubation with the secondary antibody. Finally, HSCs were observed under confocal laser scanning microscopy.

### 2.11 Cell invasion experiments

The cell invasion experiments were performed using the QCMTM 24-well Fluorimetric Cell Invasion Assay kit (ECM554, Chemicon International, USA) according to the manufacturer’s instructions using the Transwell system. The invading cells were stained with 4′,6-diamidino-2-phenylindole, and their number was determined by fluorescence and reported as the relative fluorescence units.

### 2.12 Statistical analysis

All the results are expressed as mean ± standard deviation (SD). Statistical analysis was performed using an independent Student’s *t*-test for comparison of two groups and one-way ANOVA following Tukey’s test for multiple comparisons. In both cases, differences of *P* < 0.05 were considered statistically significant.

## 3 Results

### 3.1 Activation of HSCs *in vitro*

**Figure 1:**
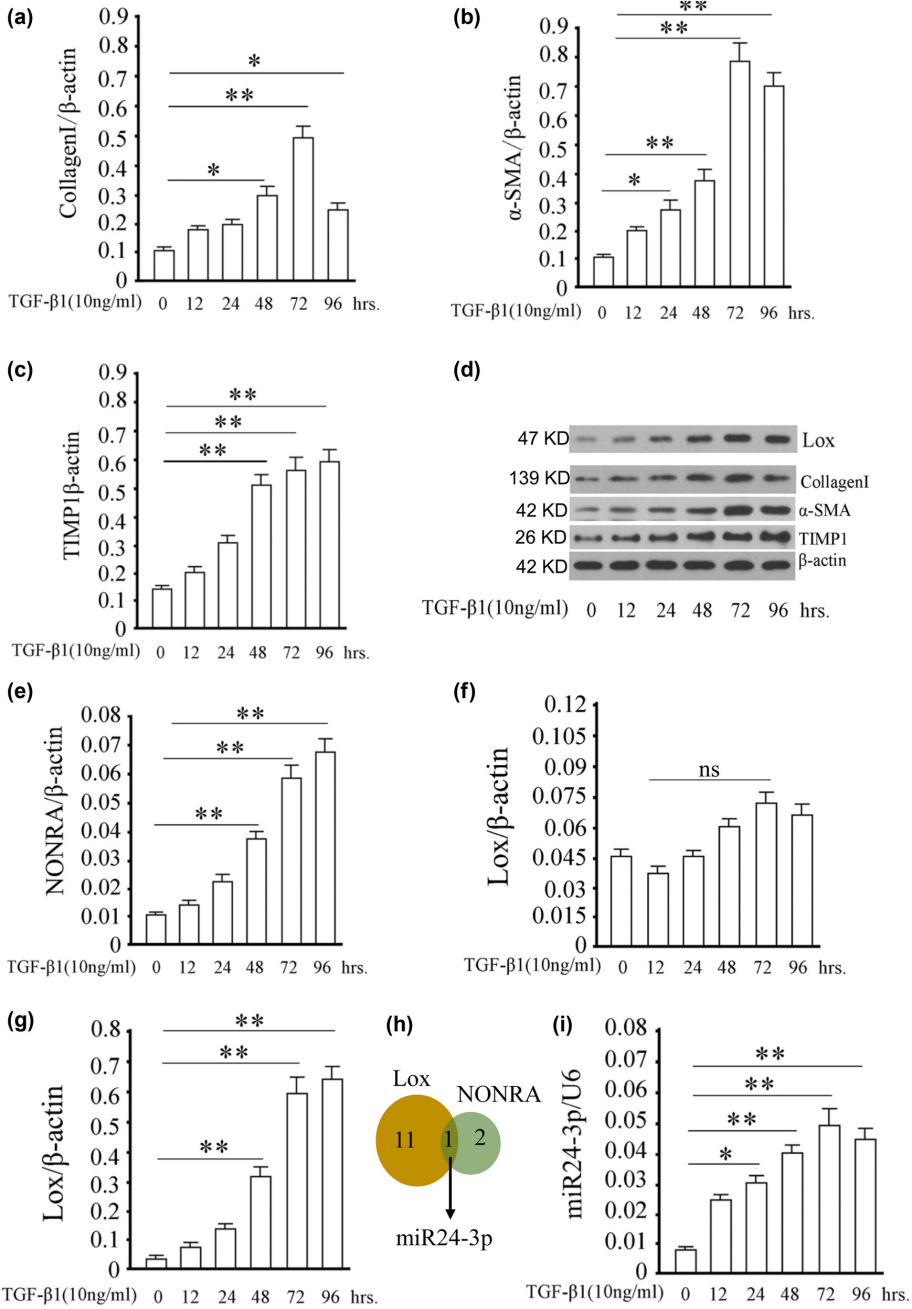
Activation of HSCs induced by TGF-β1. HSCs were induced by 10 ng/mL TGF-β1 for 0–96 h, and HSCs at different time points were collected to extract total protein. The gray statistics of western blotting of HSCs activation markers of collagen I (a), α-SMA (b), and TIMP1 (c). (d) Gel image of collagen I, α-SMA, TIMP1, and Lox of western blotting. (e) Relative expression of NONRATT013819.2 using RT-qPCR. (f) Relative expression of *lox* using RT-qPCR. (g) The gray statistics of western blotting of lox in HSCs. (h) Venn diagram of miRNAs targeted by lox and NONRATT013819.2, only miR24-3p is shared by both. (i) Relative expression of miR24-3p using RT-qPCR. *P < 0.05, *P < 0.01 vs control group (0 h induction group).

To obtain activated HSCs, 10 ng/mL TGF-β1 was used to induce HSCs and changes in the indicators at different time points were observed. After induction by 10 ng/mL TGF-β1 for 72 h, the expression of fibroblast activation markers (collagen I, α-SMA, and TIMP1) in HSCs were detected by western blotting. As a result, the protein expression of collagen I, α-SMA, and TIMP1 increased with induction time (Figure 1a–d). The protein expression of collagen I and α-SMA peaked at 72 h followed by a decrease at 96 h (Figure 1a, b, and d). TIMP1 protein expression peaked at 96 h, but there was no difference from 72 h (Figure 1c and d). These results showed that 10 ng/mL TGF-β1 induction for 72 h is the optimal induction condition for HSC activation. Thus, these induction conditions were used for further studies.

### 3.2 lncRNA NONRATT013819.2, miR24-3p, and *lox* expressions were upregulated in activated HSCs

Next, we detected the expression of NONRATT013819.2 in activated HSCs. RT-qPCR showed that the NONRATT013819.2 expression was increased over time and peaked at 96 h (Figure 1e). This increase in activated HSCs is consistent with RNA sequencing data in our previous study [6]. Since we previously revealed a positive correlation between the expression levels of NONRATT013819.2 and *lox*, we detected the expression of *lox*. Interestingly, we found that the mRNA expression of *lox* had no obvious change in the activated HSCs (Figure 1f), but the protein expression was completely consistent with the changing trend of NONRATT013819.2 (Figure 1d and g). This prompted us to speculate that ceRNA interactions contain NONRATT013819.2 and *lox*. By utilizing Targetscan7.1 (http://www.targetscan.org/), we predicted a unique miRNA that could target both NONRATT013819.2 and *lox*, namely, miR24-3p (Figure 1h). The results showed that the expression of miR24-3p was also increased over time and peaked at 72 h (Figure 1i). Therefore, NONRATT013819.2, miR24-3p, and *lox* expressions were upregulated in activated HSCs. This unusual phenomenon allows us to continue exploring the relationships among them in activated HSC.

### 3.3 NONRATT013819.2 weakened the suppression effect of miR24-3p on *lox* in a ceRNA manner

**Figure 2:**
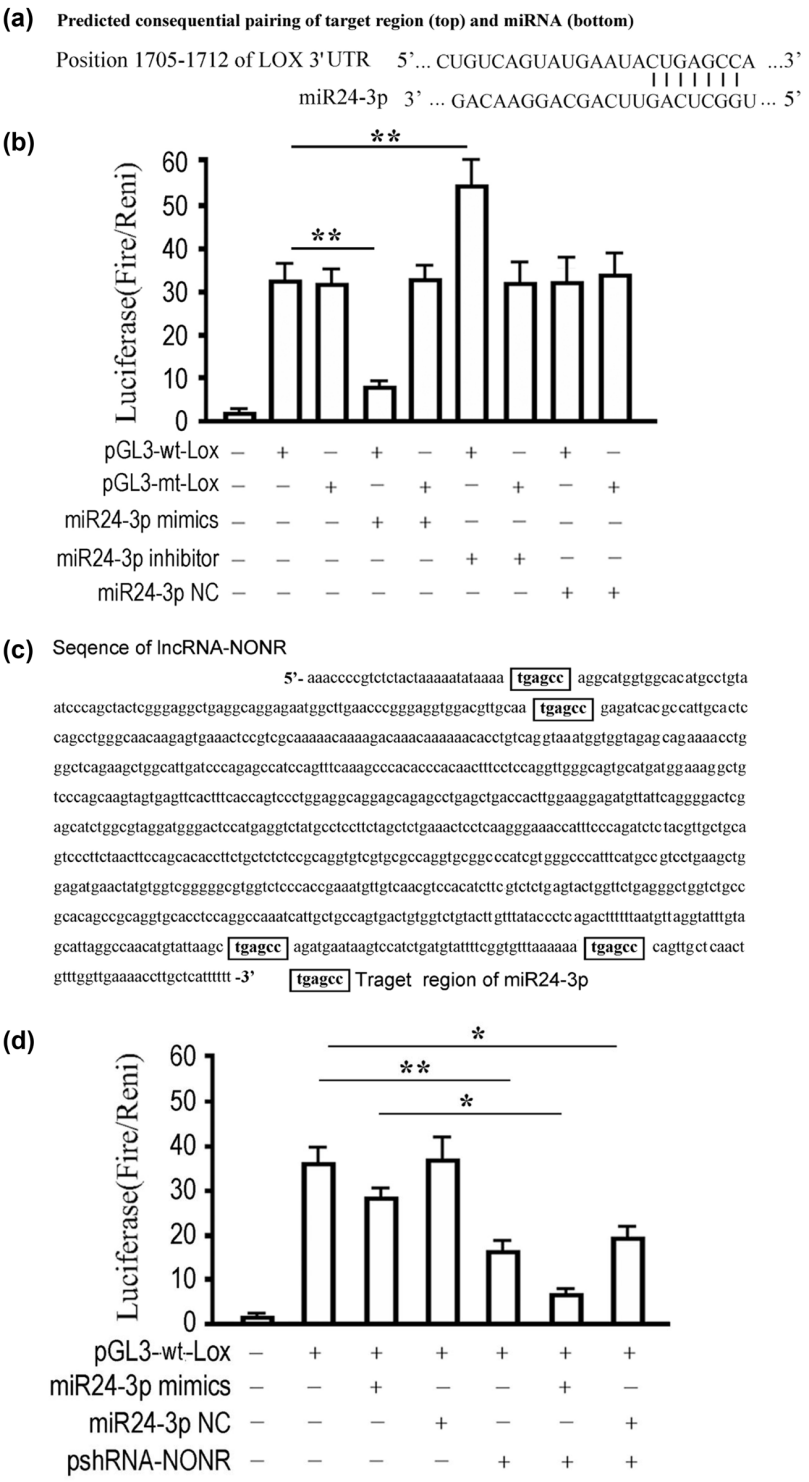
lncRNA NONRATT013819.2 competitively binds miR24-3p to reduce the binding inhibition of miR24-3p on *lox.* (a) The binding sites of miR24-3p and *lox* gene in the 3′ UTR, which were predicted with TargetScan software. (b) Dual-luciferase assay verified the interaction of miR24-3p and *lox*. The relative activity of luciferase in each group was detected at 48 h after co-transfection. (c) The analysis of binding site between NONRATT013819.2 sequence and miR24-3p. (d) In TGF-β1-activated HSCs, the effect of silencing NONRATT013819.2 on the interaction of miR24-3p with *lox* was detected by dual-luciferase assay. The relative activity of luciferase in each group was detected at 48 h after co-transfection. The results were expressed as the mean ± SD from three independent experiments, *P < 0.05, **P < 0.01.

Examination of the homology between miR24-3p and *lox* mRNA sequences showed that the six-base seed region 5′-GUAGCC-3′ is in the 3′ UTR according to the online software TargetScan (http://www.targetscan.org) (Figure 2a). Thus, an inhibitory effect of miR24-3p was inferred. Dual-luciferase assay showed that luciferase activity in the HSCs of miR24-3p mimics and miR24-3p inhibitor co-transfected with pGL3-wt-Lox was significantly lower or higher than that of pGL3-wt-Lox alone transfected group, respectively (Figure 2b). As expected, this effect could be abolished by a partial mutation in the 3′ UTR of *lox* mRNA (pGL3-mt-Lox group) (Figure 2b). These results confirmed the direct interaction between miR24-3p and *lox*.

In addition, there were also multiple miR24-3p seed regions in the NONRATT013819.2 sequence and the positions were relatively concentrated (Figure 2c). To investigate the effect of NONRATT013819.2 depletion on the luciferase activity of miR24-3p, we co-transfected with pshRNA-NONR and miR24-3p mimics. Compared with miR24-3p mimics co-transfected with pGL3-wt-Lox group, the intracellular luciferase activity of pshRNA-NONR and miR24-3p mimics co-transfected group was significantly decreased (Figure 2d). However, when miR24-3p mimics were replaced by miR24-3p NC, the aforementioned difference disappeared (Figure 2d). These results implied that there are interactions between NONRATT013819.2 and miR24-3p.

Importantly, these results suggested that silencing of NONRATT013819.2 exacerbated the suppressive effects of miR24-3p on *lox*. In addition, according to the results, silencing of NONRATT013819.2 may be responsible for the inhibitory effects of miR24-3p on *lox*.

### 3.4 Lentiviral pathway-mediated silence of NONRATT013819.2 and *lox* overexpression

**Figure 3:**
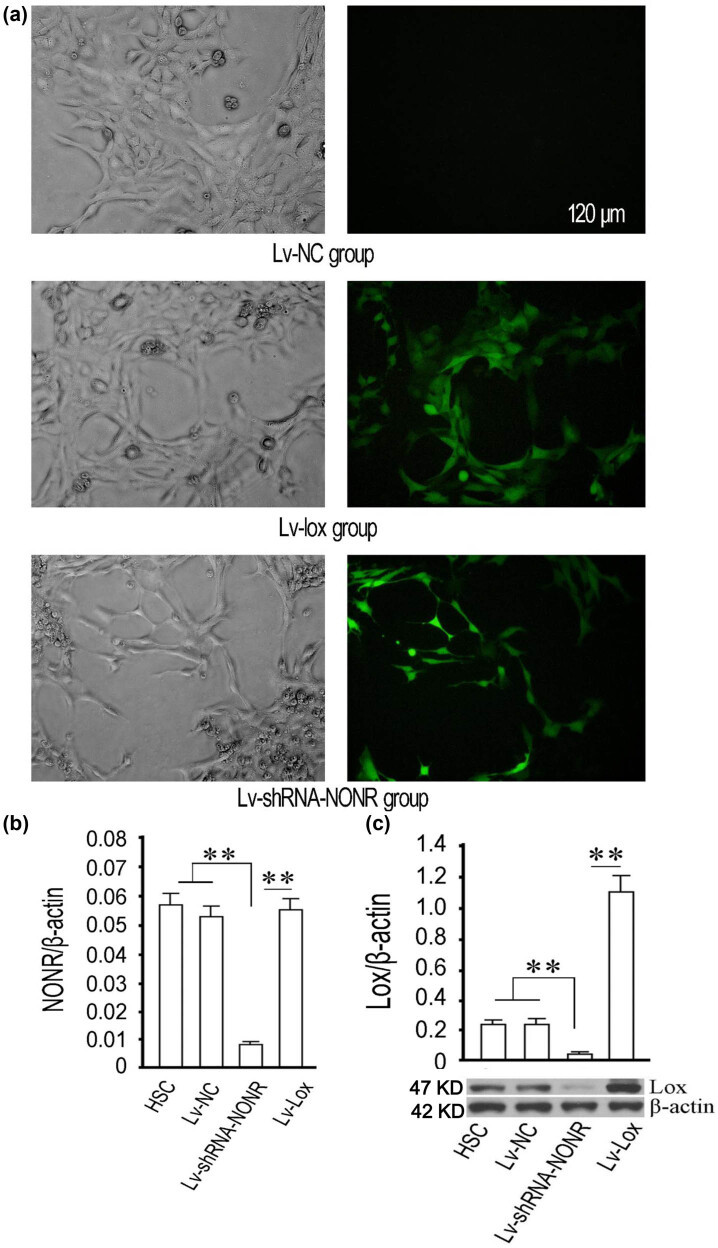
Lentiviral pathway-mediated silencing of NONRATT013819.2 and overexpression of *lox* gene in HSCs. (a) The lentivirus-mediated NONRATT013819.2 silencing was demonstrated by GFP green fluorescent protein (MOI = 10). (b) RT-qPCR was used to detect the relative amount of NONRATT013819.2. (c) Western blotting was used to detect the relative protein expression of Lox. *P < 0.05, **P < 0.01.

To explore the function of NONRATT013819.2 and *lox* on HSCs, we constructed the lentiviruses of NONRATT013819.2 silence, *lox* overexpression, and control. Next, the packaged lentiviruses were tagged Lv-shRNA-NONR, Lv-lox, and Lv-NC. The proportion of cells with GFP expression was not less than 90%, indicating that the efficiency of lentiviral infection was sufficient for further studies (Figure 3a). Compared with blank HSCs (HSC) and Lv-NC group, NONRATT013819.2 expression was significantly downregulated in the Lv-shRNA-NONR group, suggesting that the silencing efficiency was high (Figure 3b). Western blot showed that protein expression of Lox in the Lv-shRNA-NONR group was significantly decreased compared to blank HSCs group or Lv-NC group, but it was significantly upregulated in the Lv-lox group, indicating the high efficiency (Figure 3c).

### 3.5 Silencing NONRATT013819.2 inhibited biological function in TGF-β1-activated HSCs by miR24-3p/Lox

**Figure 4:**
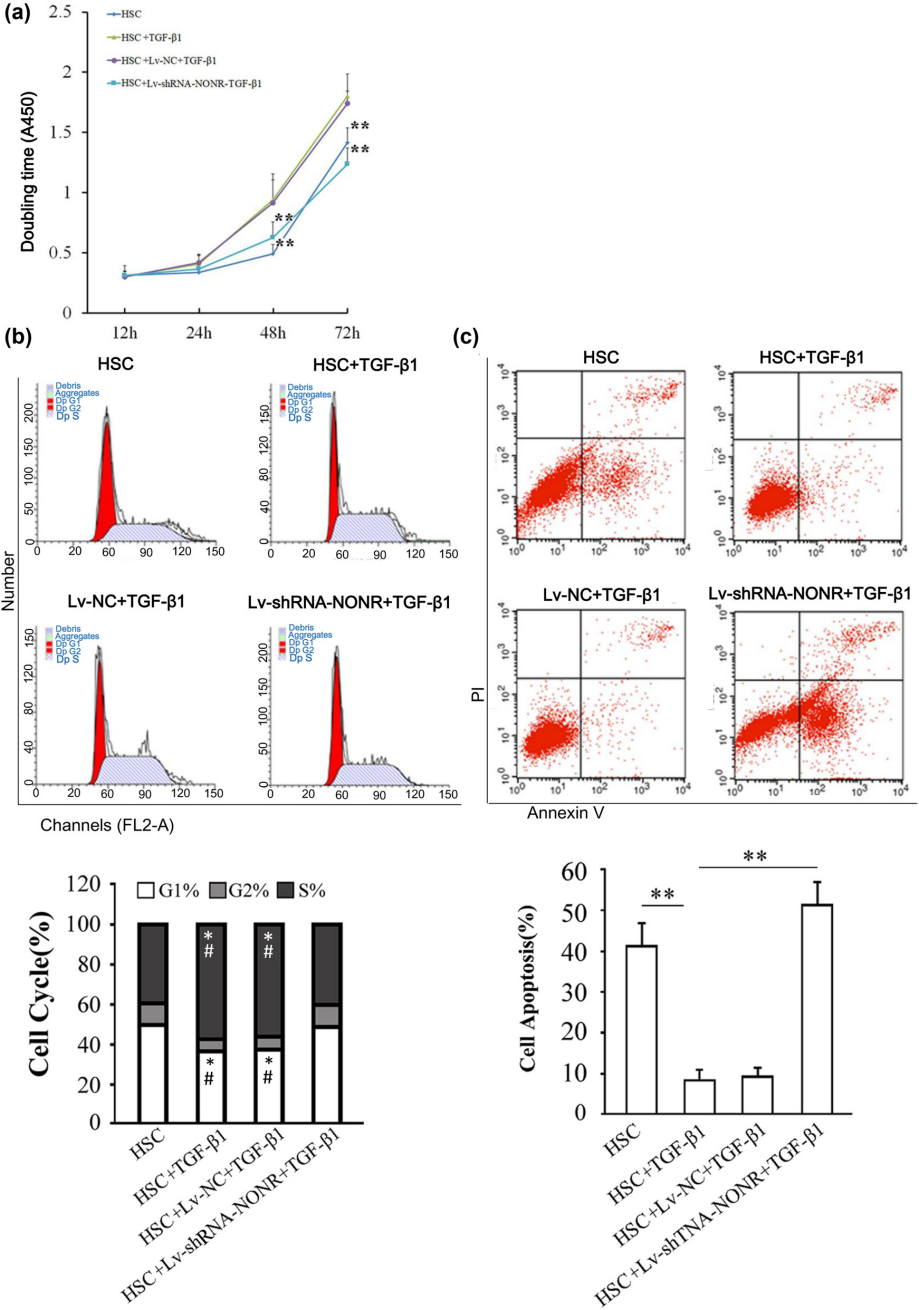
Silencing NONRATT013819.2 inhibited biological functions in TGF-β1-activated HSCs by miR24-3p/*lox*. (a) HSCs proliferation was analyzed using CCK-8 kits, and the OD value was observed at 450 nm. (b) The cell cycle of HSCs was detected by flow cytometry after silencing of NONRATT013819.2 or overexpression *lox*. *Y*-axis represents cell number. *X*-axis represents the DNA content dictated by the channel of FL2-A. Dp stands for Diploid. Both G1 and G2 are annotated in red, this is because the annotation of G1 and G2 is automatically defined by FACS Calibur without the confusion of cells in each phase. The * indicates comparison with group HSC; the # indicates comparison with group HSC + Lv-shRNA-NONR + TGF-β1. (c) The apoptosis HSCs was detected by flow cytometry after silencing NONRATT013819.2 or overexpression *lox*. The lower and upper right quadrants indicated the proportion of apoptotic cells in the early and late stages, respectively. The apoptosis rate between groups was the sum of the early and late apoptosis rates. The results were expressed as the mean ± SD from three independent experiments, # and *P < 0.05, **P < 0.01.

**Figure 5:**
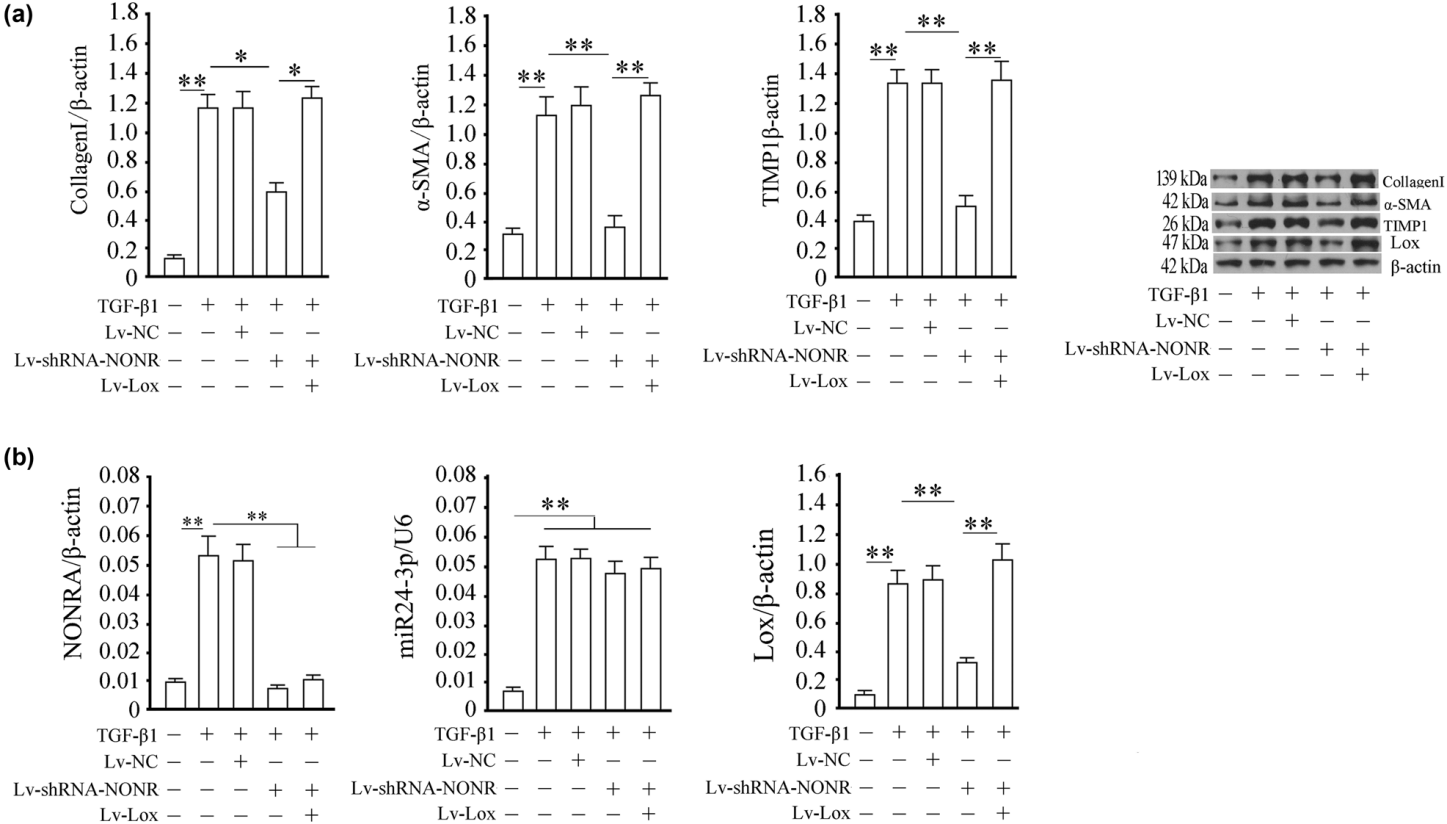
Silencing NONRATT013819.2 inhibited biological functions in TGF-β1-activated HSCs by miR24-3p/Lox. (a) The expression of activated characteristic proteins of collagen I, α-SMA, and TIMP1 was detected by western blotting. (b) The relative concentrations of NONRATT013819.2 and miR24-3p and Lox protein expression in each group of HSCs. The quantitative detection of NONRATT013819.2 and Lox was normalized by β-actin, and miR24-3p was normalized by U6. The results were expressed as the mean ± SD from three independent experiments, *P < 0.05, **P < 0.01.

Further, we assessed changes in the biological functions after silencing NONRATT013819.2 or overexpressing Lox in TGF-β1-activated HSCs. We found that TGF-β1 induction significantly increased the proliferation of HSCs (Figure 4a) and the proportion of cells that entered the S-phase (Figure 4b) and inhibited apoptosis in HSCs (Figure 4c). However, silencing NONRATT013819.2 reversed the aforementioned effects (Figure 4). These results suggested that silencing NONRATT013819.2 blocked the activation effects of TGF-β1 on HSCs. Moreover, TGF-β1-induced HSCs activation being dependent on NONRATT013819.2 was confirmed at the molecular level. As shown in Figure 5a, silencing NONRATT013819.2 inhibited the TGF-β1-induced increase in protein expressions of collagen I, α-SMA, and TIMP1. Thus, TGF-β1-induced myofibroblast transition of HSCs is in a NONRATT013819.2-dependent manner.

Subsequently, we investigated the mechanism of NONRATT013819.2-dependent myofibroblast transition of HSCs. We found that compared with NONRATT013819.2 silencing group, overexpression of Lox prominently reversed the inhibitory effects of NONRATT013819.2 silencing on protein expressions of collagen I, α-SMA, and TIMP1 (Figure 5a). These suggest that Lox is involved in the NONRATT013819.2-associated regulatory activation of HSCs. As shown in Figure 5b, silencing NONRATT013819.2 resulted in a decline in the expressions of NONRATT013819.2 and *lox*, whereas overexpression of *lox* significantly restored the expression of Lox in TGF-β1-induced HSCs. However, we observed that neither deletion of NONRATT013819.2 nor overexpression of *lox* altered the expression of miR24-3p (Figure 5b). This is consistent with the aforementioned results. Therefore, we hypothesized that this might be because NONRATT013819.2 only competitively binds to miR24-3p in a miRNA-sponge manner to weaken the inhibitory ability of miR24-3p to the target gene, *lox*, but NONRATT013819.2 does not degrade miR24-3p.

### 3.6 Silencing NONRATT013819.2 inhibited ECM reconstruction through Lox

**Figure 6:**
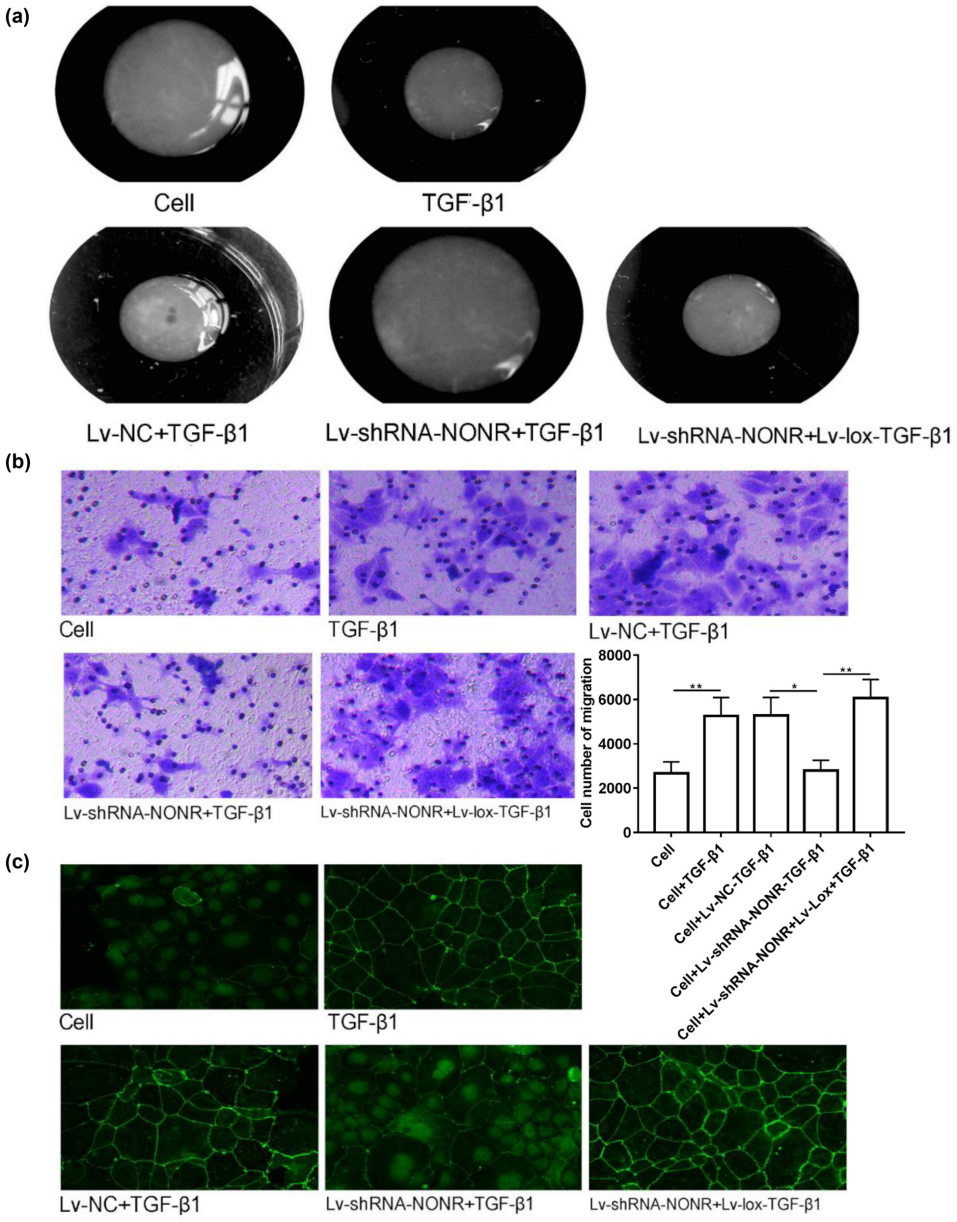
Silencing NONRATT013819.2 inhibited ECM reconstruction through Lox. (a) Contraction of hydrated collagen gels induced in HSCs. (b) The migration of HSCs was detected by the Transwell method. (c) Immunofluorescence staining of α-SMA protein in HSCs. The results were expressed as the mean ± SD from three independent experiments, *P < 0.05, **P < 0.01.

As mentioned previously, silencing NONRATT013819.2 and overexpression of *lox* affected the protein expressions of collagen I, α-SMA, and TIMP1, which play an important role in ECM reconstruction. Therefore, we then verify whether NONRATT013819.2 could change ECM reconstruction through Lox in TGF-β1-induced HSCs. The gel contraction results showed that the diameter of gels was significantly reduced in the TGF-β1-induced group, but significantly dilated after NONRATT013819.2 silencing, suggesting that silencing NONRATT013819.2 suppressed surface tension of HSCs (Figure 6a). In contrast, overexpression of *lox* exaggerated gel contraction characterized by the obviously concave surface of the gels (Figure 6a). Moreover, rescue experiments showed that silencing NONRATT013819.2 while overexpressing *lox* partially abolished gel changes caused by treatment alone (Figure 6a). Similarly, the invasion of HSCs was significantly enhanced by TGF-β1, while silencing NONRATT013819.2 inhibited this enhancement, which also could be restored by overexpression of *lox* (Figure 6b). We also detected the protein expression of α-SMA in HSCs using IF. As shown in Figure 6c, silencing NONRATT013819.2 suppressed the increased trend in α-SMA protein expression in HSCs induced by TGF-β1, which was reversed by overexpression of *lox*. Taken together, silencing NONRATT013819.2 inhibited ECM reconstruction of HSCs induced by TGF-β1 through regulating *lox*.

## 4 Discussion

HSCs go through myofibroblast transdifferentiation to acquire a migratory, myofibroblast-like phenotype is the key step in liver fibrosis. This is a multifactorial process involving excessive proliferation, apoptotic resistance, dysregulated metabolism, and remodeling of ECM [11]. All these fibrosis-inducing pathophysiological disorders demonstrate an intimate association with abnormality in various kinds of ncRNAs (e.g., miRNA, lncRNA, and circRNA) [12]. Dramatically, differential expression of lncRNAs (e.g., ATB, MALAT1, LFAR1, AS1, p21, SCARNA10, and NONRATT013819.2) usually precedes the change in the ncRNA network, which further contributes to the progression of liver fibrogenesis [6,13,14,15]. Among them, NONRATT013819.2 has been reported to have significant upregulation during HSC-to-myofibroblast transdifferentiation [6]. In this study, we proved that silencing of NONRATT013819.2 could block myofibroblastic transition of HSCs, characterized by inhibition of proliferation; the proportion of HSCs that entered S-phase; protein expressions of collagen I, α-SMA, and TIMP1; and the promotion of apoptosis, through miR24-3p/*lox* axis.

In our study, on the basis of bioinformatics predictions and the results of dual-luciferase assays, we were convinced that NONRATT013819.2 interacted with miR24-3p via base complementary pairing to further regulate the *lox* expression in a ceRNA mechanism. CeRNA means that it can competitively abolish the inhibitory effects of miRNA on a target gene and then regulates the expression of the target gene. Therefore, in the present study, the silencing of NONRATT013819.2 was uncovered to be responsible for the inactivation of miR24-3p function on *lox*. Unusually, expressions of NONRATT013819.2, miR24-3p, and *lox* were upregulated in the TGF-β1-induced HSCs. Also, either silencing NONRATT013819.2 or overexpression of *lox* could not alter miR24-3p expression. The reason may lie in the fact that the sequestering of miR24-3p by NONRATT013819.2 inevitably reduces the interactions between the miR24-3p and *lox*, which may affect the levels of free miR24-3p instead of its overall content in HSCs due to NONRATT013819.2 only sponges, but does not affect the synthesis and degradation of miR24-3p.

Growing evidence indicates that ECM composition is a critical determinant of multiple phenotypes of HSCs (i.e., proliferation, apoptosis, migration, and contraction), which are largely based on interactions between collagen and cell adhesion molecules [16]. For instance, the pathophysiological disorder of contraction of hydrated collagen gel underlies portal hypertension during the progression of liver fibrosis/cirrhosis [17]. Furthermore, an interaction of ECM (including collagens I/III, fibronectin, α-SMA, etc.) and myofibroblasts has been proved to exert important effects on the phenotypes and functions of myofibroblasts [18]. Myofibroblasts with activated phenotype induced the excessive deposition and altered the composition of ECM, which further promoted the fibrosis-inducing characteristics of myofibroblasts [18]. Therefore, in our study, increased protein expressions of collagen I, α-SMA, and TIMP1, and gel contraction were considered to be a feature of myofibroblastic transition of the HSCs.

Recently, Lox and Lox-like (LoxL) isoforms 1–3 have been shown to be involved in liver fibrosis [19]. In Cav-1^−/−^ or periostin^−/−^ mice with CCl_4_ exposure, the hepatic expression of Lox is upregulated with the exacerbation of fibrosis [19,20,21]. In contrast, administration of Lox inhibitor, β-aminopropionitrile, suppressed the accumulation of cross-linked collagens and the stiffness of collagen fibril in the ECM, accelerating fibrosis reversal after CCl_4_ withdrawal [22,23]. Mechanistically, activation of Lox family proteins (i.e., Lox and LoxLs) mediates the deoxypyridinoline and pyridinoline crosslinks in collagen, and the increase in collagen crosslinking promotes its stabilization and contributes to the irreversibility of fibrosis [24,25]. In addition, Lox and LoxLs are responsible for the tropoelastin crosslinking and polymerization of elastin that forms fibrosis-related elastic fibers [26]. Therefore, Lox promotes liver fibrosis and prevents spontaneous reversal via collagen. This conclusion is supported by our results. In the present study, overexpression of Lox synergistically promoted the activation of TGF-β1 on HSCs, characterized by increased protein expressions of collagen I, α-SMA, and TIMP1, and these effects were reversed by silencing NONRATT013819.2. Consequently, the promoting role of NONRATT013819.2 in the myofibroblastic transition of HSCs is mediated by Lox/collagen.

Numerous studies have shown that miR24-3p is involved in a wide range of human diseases, including those associated with abnormal collagen synthesis. The miR24-3p has been demonstrated to be significantly upregulated in HCV-positive hepatocellular carcinoma as compared to the control [27]. Yang et al. proved that overexpression of miR24-3p regulated collagen synthesis and skin barrier [28]. In addition, overexpression of miR24-3p elevated the protein expression of collagen type II to weaken cartilage degradation induced by IL-1β [29]. In this study, a significant increase in collagen I was observed to be accompanied by abnormal upregulation of miR24-3p, suggesting an association between them. Based on the aforementioned ceRNA theory, we concluded that NONRATT013819.2 and miR24-3p competitively binding to *lox* is the possible mechanism of collagen I responding to TGF-β1 induction.

In conclusion, TGF-β1-induced activation of HSCs resulted in expression of NONRATT013819.2 and *lox* was upregulated, and proteins of myofibroblast markers also accumulated, which may contribute to increased proliferation, apoptosis resistance, migration, and contraction of activated HSCs. NONRATT013819.2 exerts the aforementioned effects by eliminating the inhibition of *lox* expression by miR24-3p. Thus, NONRATT013819.2/miR24-3p/Lox signaling is a novel pathway involved in the myofibroblastic transition of HSCs, and we proposed a possible NONRATT013819.2-targeting strategy for the management of myofibroblastic transition and even the treatment of liver fibrosis.

## Appendix

**Table A1:** Primers used in used in this study

Gene	Function	Number gene primer (5′ → 3′)
NONRATT013819.2	RT-qPCR	F: TCCTTCGCGGGATCTGAGT
		R: CCCGGCTCGTCCCTTCT
Lox	RT-qPCR	F: GTTGAGTCCCGGATGTTATGA
		R: AGCTGGGGTTTACACTGACCT
β-actin	RT-qPCR	F: TGTACGTAGCCATCCAGGC
		R: AACCCTCATAGATGGGCACAGTG
miR-24-3p	RT-qPCR	F: GTGCTCGCTTCGGCAGCACAT
		R: GACAAGGACGACTTGACTCGGT
U6	RT-qPCR	F: GCTTCGGCAGCACATATACTAAAAT
		R: CGCTTCACGAATTTGCGTGTCAT
Lox	PCR for lentiviral vector	F: GGAATTCGCCACCATGCGTTTCGCCTGGACC
		R: CGGGATCCCTAATACGGTGAAATGGTGCAG
Lox	PCR for luciferase vector	F: GCTCTAGAAAGAAGCTCACTTCCCAAAGG
		R: GCTCTAGATTCATAAAGCAACATGAATAAG
NONRATT013819.2 siRNA	siRNA	GTAGTGAGTTCACTTTCAC
NONRATT013819.2 siRNA	shRNA-DNA	GTAGTGAGTTCACTTTCACCTTCCTGTCAGA
		GTAGTGAGTTCAC
NC-siRNA	siRNA	GTACACTATTAGTCGTCTG
NC-siRNA	shRNA-DNA	GTACACTATTAGTCGTCTGCTTCCTGTCAGA
		CAGACGACTAATAGTGTAC
miR-24-3p mimic	Luciferase assay	CCCAGUGUUCAGACUACCUGUUCtt
miR-24-3p inhibitor	Luciferase assay	GAACAGGUAGUCUGAACACUGGGtt
miR-24-3p NC	Luciferase assay	CCCAGUGUUCAGACUACCUGUUCtt

F: forward primer; R: reverse primer. Annealing temperature of NONRATT013819.2, Lox, β-actin miR-24-3p, and U6 was 60°C for RT-qPCR.

## References

[1] Dewidar B, Meyer C, Dooley S, Meindl-Beinker AN. Tgf-beta in hepatic stellate cell activation and liver fibrogenesis-updated 2019. Cells. 2019;8(11):1419. 10.3390/cells8111419. PMC6912224 31718044

[2] Zhang QD, Xu MY, Cai XB, Qu Y, Li ZH, Lu LG. Myofibroblastic transformation of rat hepatic stellate cells: The role of notch signaling and epithelial-mesenchymal transition regulation. Eur Rev Med Pharmacol Sci. 2015;19(21):4130–8. 26592839

[3] Huang YH, Chen MH, Guo QL, Chen ZX, Chen QD, Wang XZ. Interleukin-10 induces senescence of activated hepatic stellate cells via stat3-p53 pathway to attenuate liver fibrosis. Cell Signal. 2020;66:109445. 10.1016/j.cellsig.2019.109445. 31730896

[4] Ma Z, Wang YY, Xin HW, Wang L, Arfuso F, Dharmarajan A, et al. The expanding roles of long non-coding rnas in the regulation of cancer stem cells. Int J Biochem Cell Biol. 2019;108:17–20. 10.1016/j.biocel.2019.01.003. 30630112

[5] Kong Y, Huang T, Zhang H, Zhang Q, Ren J, Guo X, et al. The lncrna neat1/mir-29b/atg9a axis regulates igfbprp1-induced autophagy and activation of mouse hepatic stellate cells. Life Sci. 2019;237:116902. 10.1016/j.lfs.2019.116902. 31610195

[6] Guo CJ, Xiao X, Sheng L, Chen L, Zhong W, Li H, et al. Rna sequencing and bioinformatics analysis implicate the regulatory role of a long noncoding rna-mrna network in hepatic stellate cell activation. Cell Physiol Biochem. 2017;42(5):2030–42. 10.1159/000479898. 28803234

[7] Tay Y, Rinn J, Pandolfi PP. The multilayered complexity of cerna crosstalk and competition. Nature. 2014;505(7483):344–52. 10.1038/nature12986. PMC4113481 24429633

[8] Salmena L, Poliseno L, Tay Y, Kats L, Pandolfi PP. A cerna hypothesis: The rosetta stone of a hidden rna language? Cell. 2011;146(3):353–8. 10.1016/j.cell.2011.07.014. PMC3235919 21802130

[9] Friedman SL, Roll FJ, Boyles J, Arenson DM, Bissell DM. Maintenance of differentiated phenotype of cultured rat hepatic lipocytes by basement membrane matrix. J Biol Chem. 1989;264(18):10756–62. 2732246

[10] Guo CJ, Pan Q, Jiang B, Chen GY, Li DG. Effects of upregulated expression of microrna-16 on biological properties of culture-activated hepatic stellate cells. Apoptosis. 2009;14(11):1331–40. 10.1007/s10495-009-0401-3. 19784778

[11] Moran-Salvador E, Garcia-Macia M, Sivaharan A, Sabater L, Zaki MYW, Oakley F, et al. Fibrogenic activity of mecp2 is regulated by phosphorylation in hepatic stellate cells. Gastroenterology. 2019;157(5):1398–412 e9. 10.1053/j.gastro.2019.07.029. PMC6853276 31352003

[12] Koduru SV, Leberfinger AN, Kawasawa YI, Mahajan M, Gusani NJ, Sanyal AJ, et al. Non-coding rnas in various stages of liver disease leading to hepatocellular carcinoma: Differential expression of mirnas, pirnas, lncrnas, circrnas, and sno/mt-rnas. Sci Rep. 2018;8(1):7967. 10.1038/s41598-018-26360-1. PMC5964116 29789629

[13] Peng H, Wan LY, Liang JJ, Zhang YQ, Ai WB, Wu JF. The roles of lncrna in hepatic fibrosis. Cell Biosci. 2018;8:63. 10.1186/s13578-018-0259-6. PMC6282372 30534359

[14] Zhang K, Han Y, Hu Z, Zhang Z, Shao S, Yao Q, et al. Scarna10, a nuclear-retained long non-coding rna, promotes liver fibrosis and serves as a potential biomarker. Theranostics. 2019;9(12):3622–38. 10.7150/thno.32935. PMC6587170 31281502

[15] Zhang K, Han X, Zhang Z, Zheng L, Hu Z, Yao Q, et al. The liver-enriched lnc-lfar1 promotes liver fibrosis by activating tgfbeta and notch pathways. Nat Commun. 2017;8(1):144. 10.1038/s41467-017-00204-4. PMC5529527 28747678

[16] Puente A, Fortea JI, Cabezas J, Arias Loste MT, Iruzubieta P, Llerena S, et al. Loxl2-a new target in antifibrogenic therapy? Int J Mol Sci. 2019;20(7):1634. 10.3390/ijms20071634. PMC6480111 30986934

[17] Guo CJ, Pan Q, Xiong H, Qiao YQ, Bian ZL, Zhong W, et al. Dynamic expression of mir-126* and its effects on proliferation and contraction of hepatic stellate cells. FEBS Lett. 2013;587(23):3792–801. 10.1016/j.febslet.2013.09.047. 24140635

[18] Novo E, Cannito S, Morello E, Paternostro C, Bocca C, Miglietta A, et al. Hepatic myofibroblasts and fibrogenic progression of chronic liver diseases. Histol Histopathol. 2015;30(9):1011–32. 10.14670/HH-11-623. 25896393

[19] Ji DG, Zhang Y, Yao SM, Zhai XJ, Zhang LR, Zhang YZ, et al. Cav-1 deficiency promotes liver fibrosis in carbon tetrachloride (ccl4)-induced mice by regulation of oxidative stress and inflammation responses. Biomed Pharmacother. 2018;102:26–33. 10.1016/j.biopha.2018.03.016. 29549726

[20] Hall C, Ehrlich L, Meng F, Invernizzi P, Bernuzzi F, Lairmore TC, et al. Inhibition of microrna-24 increases liver fibrosis by enhanced menin expression in mdr2(-/-) mice. J Surg Res. 2017;217:160–69. 10.1016/j.jss.2017.05.020. PMC5760243 28602220

[21] Kumar P, Smith T, Raeman R, Chopyk DM, Brink H, Liu Y, et al. Periostin promotes liver fibrogenesis by activating lysyl oxidase in hepatic stellate cells. J Biol Chem. 2018;293(33):12781–92. 10.1074/jbc.RA117.001601. PMC6102155 29941453

[22] Liu SB, Ikenaga N, Peng ZW, Sverdlov DY, Greenstein A, Smith V, et al. Lysyl oxidase activity contributes to collagen stabilization during liver fibrosis progression and limits spontaneous fibrosis reversal in mice. FASEB J. 2016;30(4):1599–609. 10.1096/fj.14-268425. 26700732

[23] Iwasaki A, Sakai K, Moriya K, Sasaki T, Keene DR, Akhtar R, et al. Molecular mechanism responsible for fibronectin-controlled alterations in matrix stiffness in advanced chronic liver fibrogenesis. J Biol Chem. 2016;291(1):72–88. 10.1074/jbc.M115.691519. PMC4697189 26553870

[24] van der Slot AJ, Zuurmond AM, van den Bogaerdt AJ, Ulrich MM, Middelkoop E, Boers W, et al. Increased formation of pyridinoline cross-links due to higher telopeptide lysyl hydroxylase levels is a general fibrotic phenomenon. Matrix Biol. 2004;23(4):251–7. 10.1016/j.matbio.2004.06.001. 15296939

[25] Zhang Y, Ghazwani M, Li J, Sun M, Stolz DB, He F, et al. Mir-29b inhibits collagen maturation in hepatic stellate cells through down-regulating the expression of hsp47 and lysyl oxidase. Biochem Biophys Res Commun. 2014;446(4):940–4. 10.1016/j.bbrc.2014.03.037. PMC4033690 24650661

[26] Kanta J. Elastin in the liver. Front Physiol. 2016;7:491. 10.3389/fphys.2016.00491. PMC5079096 27826254

[27] Oksuz Z, Serin MS, Kaplan E, Dogen A, Tezcan S, Aslan G, et al. Serum micrornas; mir-30c-5p, mir-223-3p, mir-302c-3p and mir-17-5p could be used as novel non-invasive biomarkers for hcv-positive cirrhosis and hepatocellular carcinoma. Mol Biol Rep. 2015;42(3):713–20. 10.1007/s11033-014-3819-9. 25391771

[28] Yang Z, Duan X, Wang X, Li D, Xu Q, Xiang S, et al. The effect of q-switched 1064-nm dymium-doped yttrium aluminum garnet laser on the skin barrier and collagen synthesis through mir-24-3p. Lasers Med Sci. 2021;37:205–14. 10.1007/s10103-020-03214-9. PMC8803697 33400013

[29] Xu J, Qian X, Ding R. Mir-24-3p attenuates il-1beta-induced chondrocyte injury associated with osteoarthritis by targeting bcl2l12. J Orthop Surg Res. 2021;16(1):371. 10.1186/s13018-021-02378-6. PMC8194242 34116684

